# Guiding clinical management of patients with CNS lymphomas by minimal-invasive detection of ctDNA in cerebrospinal fluid

**DOI:** 10.1038/s41375-025-02583-w

**Published:** 2025-04-09

**Authors:** S. Weinschenk, U. Philipp, J. C. Kuehn, K. Mueller, J. Fauser, D. Boeckle, I. Gebhard, M. Hinz, N. Neidert, S. Bleul, E. M. Lauer, J. A. Mutter, S. K. Alig, D. M. Kurtz, J. Finke, R. Marks, M. Diehn, A. A. Alizadeh, P. C. Reinacher, J. Wehrle, U. Keller, D. Wolf, F. Kocher, B. Chapuy, J. Beck, M. Prinz, L. von Baumgarten, E. Schorb, J. Duyster, F. Scherer

**Affiliations:** 1 Department of Medicine I, Medical Center—University of Freiburg, Faculty of Medicine, University of Freiburg, Freiburg, Germany; 2 German Cancer Consortium (DKTK) partner site Freiburg and German Cancer Research Center (DKFZ), Heidelberg, Germany; 3 Department of Neurology, University Hospital, LMU Munich, Munich, Germany; 4 Department of Internal Medicine V (Hematology and Oncology), Comprehensive Cancer Center Innsbruck, Medical University of Innsbruck, Innsbruck, Austria; 5 Department of Hematology, Oncology, and Tumor Immunology, Charité, Campus Benjamin Franklin, University Medical Center Berlin, Berlin, Germany; 6 Department of Neurosurgery, Medical Center, University of Freiburg, Freiburg, Germany; 7 Department of Medicine, Divisions of Oncology and Hematology, Stanford University, Stanford, CA USA; 8 Department of Internal Medicine III, Ludwig-Maximilians-University (LMU) Hospital, Munich, Germany; 9 Department of Radiation Oncology, Stanford University Medical Center, Stanford, CA USA; 10 Department of Stereotactic and Functional Neurosurgery, Medical Center-University of Freiburg, Faculty of Medicine, University of Freiburg, Freiburg, Germany; 11 Fraunhofer Institute for Laser Technology (ILT), Aachen, Germany; 12 German Cancer Consortium (DKTK) partner site Charité Berlin and German Cancer Research Center (DKFZ), Heidelberg, Germany; 13 Institute of Neuropathology, Faculty of Medicine, University of Freiburg, Freiburg, Germany; 14 BIOSS Centre for Biological Signalling Studies and Centre for Integrative Biological Signalling Studies (CIBSS), University of Freiburg, Freiburg, Germany; 15 Department of Neurosurgery, University Hospital, LMU Munich, Munich, Germany; 16 German Cancer Consortium (DKTK) partner site Munich and German Cancer Research Center (DKFZ), Heidelberg, Germany

**Keywords:** Translational research, B-cell lymphoma, Genetic testing

Central nervous system lymphomas (CNSL) are aggressive extranodal Non-Hodgkin lymphomas confined to the CNS compartment and classified as large B-cell lymphomas of immune-privileged sites [1]. In contrast to other primary CNS tumors or brain metastases that are mainly treated with surgery and radiotherapy, the primary curative treatment strategy for CNSL is based on intensive immunochemotherapies followed by consolidation regimens [2]. Due to this unique therapeutic approach, stereotactic biopsy followed by histopathological assessment is the gold standard for CNSL diagnosis [2]. While neurosurgical biopsies are generally characterized by low complication rates, various clinical scenarios preclude or delay this invasive procedure and final CNSL diagnosis. For example, tumor localization in eloquent brain areas or surgical high-risk situations might result in unacceptable perioperative morbidity. Furthermore, concurrent corticosteroid or antiplatelet treatment often causes a significant delay of stereotactic procedures and might lead to indeterminate histopathological findings [3]. In these critical scenarios and due to a rapid decline of neurological recovery associated with a delay of treatment initiation in this lymphoma type, minimal-invasive identification of CNSL from cerebrospinal fluid (CSF) could have transformative impact on the clinical management of patients with unknown CNS lesions [2, 3]. This also extends to patients with suspected secondary involvement of systemic lymphomas or relapse of primary CNSL (PCNSL), who would benefit from surgery-free confirmation of diagnosis to tailor further treatment. Conventional CSF analyses such as cytopathology (CP) or flow cytometry (FC) and diagnostic magnetic resonance imaging (MRI) have shown low sensitivities and discriminative capacity to facilitate biopsy-free CNSL diagnosis, highlighting the need for more innovative technologies [3–5]. Previous research studies have demonstrated reliable detection of the lymphoma-specific *MYD88* L265P mutation in circulating tumor DNA (ctDNA) from CSF of CNSL patients and showed a potential treatment-guiding effect in anecdotal cases or in small case series [3, 6–10]. However, the value of a liquid biopsy approach for surgical planning and treatment management in large patient cohorts and daily clinical practice has never been prospectively explored. Therefore, we here developed and implemented a sensitive and highly specific digital droplet PCR (ddPCR) assay in a clinical laboratory environment for minimal-invasive detection of *MYD88* L265P from CSF and evaluated its impact on clinical management in a cohort of 182 patients.

We first underwent an extensive approval process by the national accreditation body of the Federal Republic of Germany in our laboratory at the University Medical Center Freiburg to develop and validate the ddPCR assay as a laboratory-developed test (LDT). The assay settings, performance parameters, and technical results that led to the approval of the LDT for its use in clinical routine are described in detail in the Supplementary Information (Supplementary Fig. S1, Supplementary Tables 1-6). The development process revealed a limit of blank of 0.5 detected mutant copies per mL sample volume and a limit of detection of 0.05% mutant allele frequency (AF) as key parameters that were applied to all subsequent analyses to determine the presence of *MYD88* L265P in body fluids. Then, the performance of the assay was validated in an independent research study cohort of 128 patients with contrast-enhancing brain lesions and verified histopathological diagnoses to evaluate its diagnostic performance in both CSF (*n* = 77) and plasma (*n* = 91) (Supplementary Fig. S2). The assay showed a sensitivity of 67% in CSF and 37% in plasma, with a specificity and positive predictive value (PPV) of 100% (Supplementary Fig. S3, Supplementary Table 7). All further details of the validation process are described in the Supplementary Information.

After implementation in our clinical laboratory environment, we applied the *MYD88* L265P ddPCR assay to 205 CSF samples collected from 182 hospitalized patients and submitted to our laboratory between 01/2022 and 06/2024 (Supplementary Fig. S4A, Supplementary Table 8). CSF samples were obtained at the discretion of the treating physician and submitted from hospitals of the University Medical Center Freiburg (*n* = 85) and 20 external referral centers (*n* = 120). The number of CSF submissions steadily increased from a median of 8 samples per quarter in 2022 to a median of 46.5 samples per quarter in 2024 (Fig. 1A). Submitted CSF volumes ranged from 0.5 to 10 mL, with a median of 2.9 mL (Fig. 1B). We reported the results of the *MYD88* L265P ddPCR assay to treating physicians with a median turnaround time of 5 days and detected the hotspot variant in 68 CSF samples (33%, Fig. 1C-D, Supplementary Table 8). The mutant AFs ranged from 0.055% to 25.2% in ctDNA-positive CSF samples, with a median of 0.55% (Fig. 1E).

**Fig. 1:**
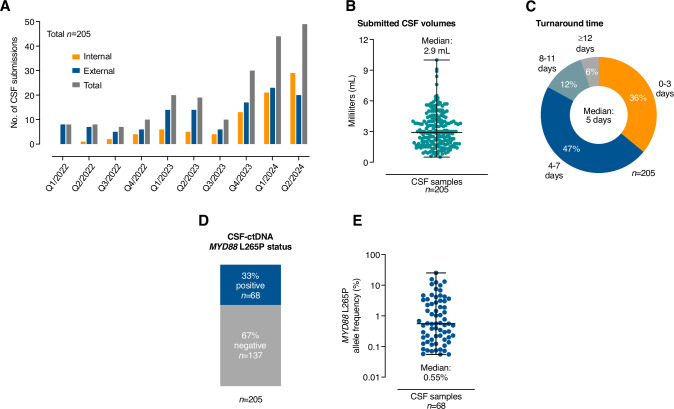
*MYD88* L265P ddPCR assay application in clinical practice. **A** Number of CSF specimens submitted from internal (orange) and external (blue) sites for routine *MYD88* L265P testing (grey: total submissions), shown by quarter from quarter 1 in 2022 until quarter 2 in 2024. **B** Scatter plot depicting submitted CSF volumes from all 205 samples in milliliters (mL). Lines highlight the median and range. **C** Pie chart showing the distribution of turnaround times in days for *MYD88* L265P analyses. **D** Bar graph depicting the fraction of samples with positive (blue) and negative (gray) CSF-ctDNA results. **E** Scatter plot highlighting CSF-ctDNA allele frequencies for all CSF analyses. Lines show the median and range. CSF, cerebrospinal fluid; Q, quarter; mL, milliliters; ctDNA, circulating tumor DNA.

127 patients, providing 143 CSF samples, participated in our observational study, facilitating the evaluation of clinical, pathological, and radiological information as well as the assessment of clinical implications following CSF-ctDNA analyses (Supplementary Fig. S4A, Supplementary Table 9). Reasons for CSF submission in the observational study included the presence of an unclear brain lesion with CNSL as differential diagnosis ([1], *n* = 49, 34%) and either surgical high-risk situation due to patient frailty or localization in an eloquent brain region ([1a], *n* = 25, 17%), delay of surgery due to concomitant steroid/antiplatelet treatment ([1b], *n* = 8, 6%), or the lack of a definitive diagnosis after biopsy and histopathological assessment ([1c], *n* = 16, 11%), a suspected secondary brain manifestation of systemic lymphoma either as occult involvement ([2a], *n* = 35, 24%), synchronous involvement ([2b], *n* = 6, 4%), metachronous SCNSL relapse ([2c], *n* = 15, 10%), or a suspected PCNSL relapse ([2 d], *n* = 8, 6%), and the request to profile CSF-ctDNA as a monitoring biomarker in patients with known CNSL ([3], *n* = 30, 21%) (Fig. 2A). *MYD88* L265P was detected in 52 of 143 CSF samples (36%) from patients participating in the observational study (Supplementary Fig. S4A). In 31 CSF-ctDNA positive cases (31/52, 60%), the ddPCR results directly guided or helped guiding further treatment and surgical management (Fig. 2B-D). Minimal-invasive identification of *MYD88* L265P obviated the need for neurosurgical biopsies or accelerated the diagnosis of CNSL in 19 patients (19/52, 37%) and led to the initiation of CNSL-specific treatment in 25 cases (25/52, 48%) (Fig. 2B-D). Specifically, 33 patients did not undergo surgery or biopsies were delayed due to surgical high-risk situations ([1a,1b], Fig. 2B). Positivity of CSF-ctDNA (*n* = 9) eliminated the need for neurosurgical interventions in 78% (7/9) of these patients and was treatment-guiding in 56% of cases (5/9). Two patients received an accelerated diagnosis of CNSL by 6 and 11 days. In 16 patients, histopathological assessment following stereotactic biopsies revealed no definitive diagnosis ([1c], Fig. 2C). *MYD88* L265P was detected in 6 of these patients (38%), leading to CNSL-specific treatment in 83% (5/6) of cases without further pathological confirmation and supported the diagnosis of a suspected cerebral amyloidoma from Waldenstrom macroglobulinemia (Fig. 2C). Finally, we received 64 CSF samples from patients with suspected CNS involvement of systemic lymphomas or suspected PCNSL relapse and found the CNSL-specific hotspot variant in 18 cases (28%, [2], Fig. 2D). These positive findings contributed to surgical planning and obviated stereotactic biopsies in 50% (9/18) of patients, while 15 (83%, 15/18) received CNSL-specific treatment following CSF-ctDNA profiling (Fig. 2D).

**Fig. 2:**
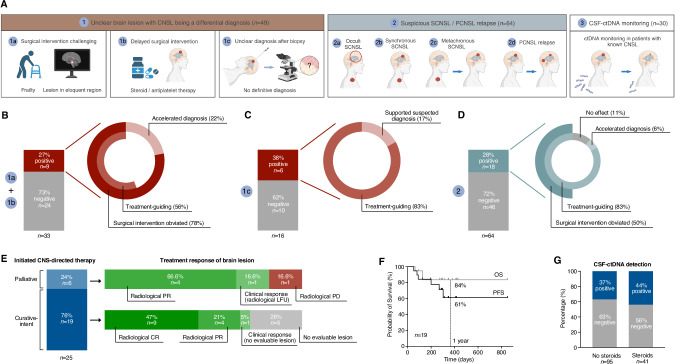
Implications of CSF-ctDNA analyses for clinical management of patients. **A** Overview and graphical depiction of the reasons for CSF submissions for routine *MYD88* L265P testing in CSF. Left [1]: Patients with unclear brain lesions and CNSL as differential diagnosis and either high-risk situation due to patient frailty or tumor lesions in eloquent brain regions [1a], delay of surgical intervention due to concomitant corticosteroid or antiplatelet treatment [1b], or unclear diagnosis after surgical intervention [1c]. Middle [2]: Patients with suspected secondary CNS involvement of systemic lymphomas either as occult [2a], synchronous [2b], or metachronous [2c] manifestation or suspected PCNSL relapse [2 d]. Right [3]: CSF submitted from patients with known CNSL for CSF-ctDNA monitoring. Bar plots and pie charts showing the fraction of patients with CSF-ctDNA positivity (red) and negativity (grey) among patients in categories [1a,b] (**B**) and [1c] (**C**) and the effects of CSF-ctDNA detection for surgical planning and treatment initiation. **D** Bar plot and pie chart showing the fraction of patients with CSF-ctDNA positivity (tourquoise) and negativity (grey) among patients in categories [2] and the effects of CSF-ctDNA detection for surgical planning and treatment initiation. **E** This panel highlights patients who received CNSL-specific treatment based on CSF-ctDNA positivity. The left bar plot shows the fraction of patients obtaining either curative-intent (dark blue) or palliative (light blue) CNS-directed therapies. Right: These two bar plots highlight the response to these therapies. Dark green: radiological CR, light green: radiological PR, lightest green: clinical response (radiologically LFU or no evaluable brain lesion), grey: no evaluable brain lesion by MRI due to occult involvement, red: radiological PD. **F** Kaplan-Meier analysis of PFS (black) and OS (grey) in patients undergoing curative-intent therapy based on positive CSF-ctDNA results. The dotted line marks the 1-year time point. **G** Bar charts revealing the proportion of CSF samples being ctDNA positive (blue) or negative (grey) in patients with no corticosteroids (left) or with corticosteroid treatment (right) prior to and during lumbar puncture. CNSL, central nervous system lymphoma; SCNSL, secondary CNSL; PCNSL, primary CNSL; CSF, cerebrospinal fluid; ctDNA, circulating tumor DNA; CR, complete response; PR, partial response; PD, progressive disease; LFU, last follow-up; PFS, progression-free survival; OS, overall survival.

CNS-directed therapies were initiated in 25 patients with the support of our analyses. While 6 patients (24%) received palliative treatment, 19 patients (76%) underwent curative-intent immunochemotherapies that were mostly based on high-dose methotrexate (Fig. 2E, Supplementary Table 9). 94% of patients with an evaluable CNS lesion by MRI responded to therapies (*n* = 17), while two patients with no radiological follow-up or evaluable brain lesion showed a clinical response. Yet, one patient experienced a rapid progression following palliative rituximab and radiotherapy initiated based on CSF-ctDNA results (Fig. 2E). Supplementary Fig. S4B-E highlight four representative cases in which CNSL-specific therapies were applied based on minimal-invasive genotyping from CSF, leading to clinical responses of their brain lesions. One-year progression-free survival and overall survival of patients undergoing curative-intent treatment were 61% and 84%, respectively (Fig. 2F, median follow-up: 312 days).

Finally, we explored the influence of corticosteroid treatment on CSF-ctDNA detection rates and concentrations. We found no association between CSF-ctDNA concentrations and cumulative corticosteroid doses or differences in *MYD88* L265P detection rates between patients who received corticosteroids prior to the lumbar puncture and those who did not undergo corticosteroid therapy (Fig. 2G, Supplementary Fig. S5).

In conclusion, we demonstrate that minimal-invasive identification of CNSL by ctDNA profiling from CSF can effectively guide treatment and surgical management in daily clinical practice for a substantial subset of patients with unknown CNS lesions in challenging clinical situations. The regulatory development and validation processes of our LDT were optimized for high specificity to minimize false-positive results and misclassification, revealing a specificity and PPV of 100% in our study. However, *MYD88* L265P mutations have been associated with other brain diseases and clinical conditions, including patients with Bing-Neel syndrome or individuals with CHIP or IgM-MGUS, introducing a potential risk of misclassification [11, 12]. Furthermore, blood contamination of CSF in patients with systemic DLBCL could theoretically be another source of false-positive results. While these scenarios are unlikely, they require a careful assessment of the results by the treating physician and interpretation in the specific clinical context. To provide further clarity, we have included a dedicated paragraph in the Supplementary Information that explores these considerations in greater detail, addressing each specific scenario individually.

## Supplementary information

Supplementary Information

Supplementary Tables

## Data Availability

All relevant data including anonymized clinical and demographic data on patients considered in this study, as well as the mutational status of the *MYD88* L265P gene locus are fully provided in the Supplementary Tables. No other mutational data, genomic features, software, mathematical models, or codes were collected or used in this study. Reasonable requests for any additional information or material will be reviewed by the senior author (FS) to determine whether they can be fulfilled in accordance with privacy restrictions.
