# Supplementary material for: Guiding clinical management of patients with CNS lymphomas by minimal-invasive detection of ctDNA in cerebrospinal fluid
Source: Leukemia. 2025 Apr 9;39(5):1265–8. doi: 10.1038/s41375-025-02583-w (PMC12055572; doi:10.1038/s41375-025-02583-w)
Supplement: Supplementary file 1 — Supplementary Information [file 41375_2025_2583_MOESM1_ESM.pdf]

Supplementary Information for

Guiding clinical management of patients with CNS lymphomas by minimal-invasive detection of circulating tumor DNA in cerebrospinal fluid

S. Weinschenk, U. Philipp, J. C. Kuehn, K. Mueller, J. Fauser, D. Boeckle, I. Gebhard, M. Hinz, N. Neidert, S. Bleul, E. M. Lauer, J. Mutter, S. Alig, D. M. Kurtz, J. Finke, R. Marks, M. Diehn, A. A. Alizadeh, P. C. Reinacher, J. Wehrle, U. Keller, D. Wolf, F. Kocher, B. Chapuy, J. Beck, M. Prinz, L. von Baumgarten, E. Schorb, J. Duyster, and F. Scherer^

^ Corresponding author. Email: florian.scherer@uniklinik-freiburg.de

Table of contents

Supplementary Methods

Supplementary Discussion

Supplementary Tables – attached separately as Excel file

Supplementary Table 1. Primer, probes, and PCR conditions used for the design of the *MYD88* L265P ddPCR assay.

Supplementary Table 2. Determining limit of detection for the *MYD88* L265P ddPCR assay.

Supplementary Table 3. Determining limit of blank for the MYD88 L265P ddPCR assay in 21 plasma samples from healthy controls.

Supplementary Table 4. Determining analytical specificity by analyzing 13 plasma samples from elderly healthy individuals.

Supplementary Table 5. Plasma samples from CNSL patients with known *MYD88* L265P status used for assessment of inter-assay reliability.

Supplementary Table 6. Plasma samples from CNSL patients used for the assessment of intra-assay reliability.

Supplementary Table 7. Overview and characteristics of CSF / plasma samples from patients included in the independent validation cohort.

Supplementary Table 8. Overview and characteristics of CSF samples submitted for routine ctDNA testing by the *MYD88* L265P ddPCR assay.

Supplementary Table 9. Overview and characteristics of CSF samples and patients participating in the observational study.

Supplementary Figures

Supplementary Fig. S1. Performance parameters of the development workflow for the *MYD88* L265P ddPCR assay.

Supplementary Fig. S2. Independent validation of the *MYD88* L265P ddPCR assay.

Supplementary Fig. S3. ddPCR assay performance in the independent validation cohort.

Supplementary Fig. S4. Clinical implementation of the *MYD88* L265P ddPCR assay and its implications for individual patient management.

Supplementary Fig. S5. Association of the cumulative steroid dose and *MYD88* L265P AFs.

Supplementary Methods

Patient cohorts and ethics statements

Assay development

For the design and development of the *MYD88* L265P digital droplet PCR (ddPCR) assay (see below), plasma samples from patients with central nervous system lymphoma (CNSL, *n*=21) and healthy individuals (*n*=34) were used. These patients and healthy individuals participated in a research study conducted at the University Medical Center Freiburg, Germany (DRKS00015307), which was approved by the local ethics committee according to the Declaration of Helsinki and explores the role of circulating tumor DNA (ctDNA) in blood plasma and cerebrospinal fluid (CSF) as a biomarker in patients with CNSL(1).

Assay validation

For validation of the ddPCR assay in an independent patient cohort, a total of 77 CSF and 91 plasma samples from patients included in two research studies were used: The research study conducted at the University Medical Center Freiburg (Germany) as described above (DRKS00015307) and a study conducted at the University Hospital of the Ludwigs-Maximilian-University (LMU) Munich (Germany, Nr. 22-0008), both approved by the ethics committees according to the Declaration of Helsinki. Characteristics of patients participating in the independent validation cohort are presented in the main manuscript and Supplementary Tables. Importantly, plasma samples used in the independent validation cohort were independent from those analyzed for assay development (see above). In addition, 49 tumor samples from

CNSL patients with positive *MYD88* L265P status were included in this work. These tumor samples were obtained from patients participating in the research study conducted at the University Medical Center Freiburg (Germany) as described above (DRKS00015307).

Assay implementation and applications

The ddPCR assay was applied to 205 CSF samples from 182 hospitalized patients and submitted from 21 internal and external centers for *MYD88* L265P testing in our clinical laboratory. A subset of 127 patients participated in an observational study (DRKS00034686) that was approved by the local ethics committee according to the Declaration of Helsinki and allowed the assessment of all patient-related and disease-related clinical information and parameters for this analysis.

<u>Processing of CSF, plasma, and tumor samples</u>

Samples obtained within the research study conducted at the University Medical Center Freiburg, Germany (DRKS00015307)

Blood samples were collected in 10 mL EDTA tubes (Sarstedt) and centrifuged in two steps: 1) at 800 x g for 10 min to separate cells from plasma, and 2) at 1000 x g for 10 min. Plasma and plasma-depleted whole blood (PDWB) were transferred to 1.8–2 mL tubes and stored at -80° C. CSF samples were centrifuged at 800 x g for 10 min (4° C) and separated into the cell-free DNA-containing supernatant and the cellular pellet fraction. The pellet was subsequently resuspended with 500 μl PBS. The supernatant and pellet were stored at -80° C. Plasma and CSF samples were further processed in

our laboratory following the same protocols as previously described(1). Specifically, the QIAamp Circulating Nucleic Acid Kit (Qiagen) was used to isolate cell-free DNA (cfDNA) from plasma or CSF supernatant according to the manufacturer's instructions. Genomic DNA (gDNA) from the cellular CSF fraction was isolated using the QIAamp DNA Mini Kit (Qiagen) according to the manufacturer's instructions. Isolated genomic DNA from the cellular CSF fraction was shorn as previously reported(1). Then, cfDNA from the CSF supernatant and shorn gDNA from the CSF pellet were pooled after isolation for subsequent analyses, as described before to increase sensitivity(1-3). Isolated cfDNA from plasma and pooled DNA from CSF were then used for subsequent analyses described below.

Tumor samples were either obtained formalin-fixed paraffin-embedded (FFPE) tissue or fresh / fresh-frozen specimens. Tumor genomic DNA was isolated from FFPE blocks or slides using the AllPrep DNA/RNA FFPE Kit (Qiagen) and from fresh / fresh-frozen samples with the AllPrep DNA/RNA Mini Kit (Qiagen) und used for subsequent analyses.

Samples obtained within the research study conducted at the University Hospital of the Ludwigs-Maximilian-University (LMU) Munich (Germany) (Nr. 22-0008)

CSF samples were centrifuged at 200 x g for 10 min and the supernatant was aliquoted into 2mL tubes and stored at -80° C. The CSF samples were shipped to Freiburg on dry ice and further processed in our laboratory to isolate cfDNA from the supernatant for subsequent analyses as described above.

CSF submissions for routine MYD88 L265P testing

CSF specimens were collected native without the addition of stabilizing reagents from internal and external centers for routine analysis of *MYD88* L265P and shipped at room temperature to our clinical laboratory without the requirement of any processing at external sites. Median time from collection to arrival was 1 day (IQR: 1 day, average: 2 days). It was communicated with the treating physician to submit an amount of at least 3 mL CSF. However, the ddPCR analyses were performed regardless of the CSF volume due to the often critical clinical situations. In cases with a submitted CSF volume of less than 1 mL, we report the result with a note indicating that it should be interpreted with caution due to the low CSF volume. After arrival, the CSF was immediately centrifuged at 800 x g for 10 min and separated into the cfDNA-containing supernatant and the cellular pellet fraction. The pellet was subsequently resuspended with 200 μl RLT plus buffer. If possible, supernatant and resuspended cell pellet were immediately processed or, if this was not possible, stored at -80° C. Isolation of cfDNA from the supernatant was performed using the QIAamp Circulating Nucleic Acid Kit (Qiagen) and gDNA from the cell pellet was isolated with the QIAamp DNA Mini Kit (Qiagen) according to the manufacturer's instructions as described above. After isolation, cfDNA from the CSF supernatant and gDNA from the CSF pellet were pooled after isolation for subsequent analyses. Notably, we did not quantify DNA after isolation from CSF to avoid DNA loss for subsequent analyses.

<u>Development and approval process of the *MYD88* L265P ddPCR assay</u>

We developed a digital droplet PCR (ddPCR) assay as a laboratory-developed test (LDT) for the detection of *MYD88* L265P in circulating tumor DNA (ctDNA) of body fluids. The development of this ddPCR assay was conducted in a clinical laboratory at the Department of Medicine I (Hematology, Oncology, and Stem Cell Transplantation) of the University Medical Center Freiburg (Germany) that was accredited by the Deutsche Akkreditierungsstelle (DAkkS, 'National accreditation body of the Federal Republic of Germany', D-ML-13134-07-00) for the usage of LDTs that test the presence of single mutations in ctDNA of body fluids by ddPCR according to the DIN ISO 15189:2014-11 criteria.

In a first step, we designed Locked nucleic acid (LNA) probes and corresponding primer pairs for the detection of *MYD88* L265P (genomic coordinate in hg19: chr3:38182641, also annotated as *MYD88* L273P in certain annotation tools) using the Beacon Designer v.8.20 software (Premier Biosoft, Palo Alto, CA, USA), optimized for its use on cfDNA from body fluids. Primers and probes were manufactured by Integrated DNA Technologies (IDT, Leuven, Belgium). Mutant (Mut) probes were labeled with 6-carboxyfluorescein (FAM), wildtype (WT) probes were labeled with hexachlorofluorescein (HEX). Primer and probe sequences are listed in **Supplementary Table 1**. For each ddPCR run, 7 µL of the DNA template (i.e. either gBlock [see below], isolated DNA from CSF, or isolated DNA from plasma), 1.1 µL of probes (250 nM final concentration each), and 0.22 µL of primers (forward and reverse, 900 nM final concentration each) were added to 11 µL of ddPCR Supermix for Probes (Bio-Rad; total of 22 µL). Next, 20 µL of this mixture was automatically transferred into a cartridge of an Automated Droplet Generator (QX100/200™, Bio-Rad) along with 70 µL of generation oil. After droplet generation, the reactions were

transferred into a 96-well PCR plate (Bio-Rad). PCR was performed using a C1000 Touch thermal cycler (Bio-Rad). Droplet fluorescence was then measured using a QX100/200™ Droplet Reader (Bio-Rad) and the results were analyzed using QuantaSoftware v1.7.4.0917 (Bio-Rad). Optimal PCR conditions and temperature gradients were determined for the assay (**Supplementary Table 1**). Within each run, one negative control (wildtype only), one positive control, and one control without a template (non-template control, NTC) were included.

After the design of the *MYD88* L265P ddPCR assay, it was approved for routine use following the DIN ISO 15189:2104-11 criteria of the DAkkS (D-ML-13134-07-00). For the approval process, several performance criteria were determined:

Limit of detection / Analytical sensitivity and linearity

To determine the limit of detection (LOD) for the *MYD88* L265P ddPCR assay, serial dilutions of respective recombinant mutant DNA fragments (gBlock, IDT, USA) in the presence of human genomic DNA (Roche Diagnostics, Mannheim, Germany) were performed, while the relative mutant allele concentrations were set to 1:20, 1:40, 1:100, 1:200, 1:400, 1:800, 1:1,000, 1:2,000, and 1:10,000 and run in at least 4 wells. Human genomic DNA without recombinant mutant DNA fragments were included as WT controls. Significant mutant DNA detection was determined by comparing the mutant DNA samples (average of the 4 wells) with the WT control using an unpaired two-tailed t-test. The LOD was then calculated as the ratio of mutant copies vs. the total number of copies from the highest dilution that was significantly above the background, for our *MYD88* L265P assay 0.05% (**Supplementary Table 2,**

Supplementary Fig. S1A). In addition, linearity was assessed by correlating measured and expected *MYD88* L265P AFs of the dilution series (p<0.0001, r=0.99, **Supplementary Fig. S1A**).

Limit of blank

To determine the limit of blank (LOB), the *MYD88* L265P ddPCR assay was run using cfDNA extracted from blood plasma of young 21 healthy individuals known to be *MYD88* L265P negative (median age: 26 years, range: 19-32 years, **Supplementary Table 3**) and calculated as follows: LOB = mean of mutant copies per $well_{HealthyControls}$ + 3 × ($SD_{Mean\ of\ mutant\ copies\ per\ well}$). The LOB for our assay was 0.2 copies per well or, normalized to the sample volume, 0.5 copies per milliliters (mL) sample volume (**Supplementary Table 3**). Notably, in consultation with the DAkkS, we here decided to apply a strict mathematical formula that differs slightly from the commonly used formula for LOB (mean(blank) + 1.645 × (SD(blank))) to ensure high specificity of our approach(4). Further, as strongly recommended by the DAkkS, we used plasma from healthy and nondiseased young subjects as blank samples for the assessment of LOB. These plasma samples tested negative for *MYD88* L265P by targeted next-generation sequencing (NGS, see below), i.e. did not contain any mutant molecules when assessed with an alternative sensitive technology, and were representative of the nature of the samples to be tested (in contrast to water that lacks comparable biological characteristics).

Collectively, a sample was considered positive if the detected mutant copies were above the LOB of 0.5 mutant copies per mL sample volume and if the detected mutant AF was above the LOD of 0.05%. Further, a total of 50 DNA copies (mutant + wildtype) per mL sample volume was required to faithfully report the results of our analyses. In the case of less than 50 copies per mL sample volume, a negative result was reported.

Analytical specificity

Since our approach requires optimal specificity for robust identification of central nervous system lymphoma (CNSL), we additionally assessed the analytical specificity of the *MYD88* L265P ddPCR assay in an independent cohort of elderly healthy individuals (*n*=13) with a median age of 56 years, applying the above defined LOD and LOB (**Supplementary Table 4**)(5). In these healthy control samples, no false-positive background errors were found, revealing an analytical specificity of 100%.

Inter-assay reliability

To assess inter-assay reliability, 13 plasma samples from lymphoma patients with known *MYD88* L265P mutation status were repeatedly analyzed from two distinct operators on two different days, applying the LOD and LOB of our assay (**Supplementary Table 5**). For this performance parameter, no divergent results between the operators and a Pearson correlation coefficient (PCC) of at least 0.9 for the correlation of AFs between both measurements were considered acceptable. **Supplementary Fig. S1B,C** highlights that the results for ctDNA positivity (*n*=7) and ctDNA negativity (*n*=6) were 100% concordant and that there was a significant

correlation of mutant AFs with a PCC of 0.99 ($p<0.0001$), demonstrating high inter-assay reliability.

Intra-assay reliability

Intra-assay reliability was assessed by comparing the number of DNA copies detected across single wells within the assay. We investigated four technical replicates of eight plasma samples from lymphoma patients to quantify the sum of mutant and WT copies per well. We then calculated the variation coefficient (in %) of these replicates, defining a coefficient of less than 20% for each individual sample as acceptable for our assay. **Supplementary Fig. S1D** demonstrates high intra-assay reliability with a maximum variation coefficient of 4.43% (**Supplementary Table 6**).

Accuracy

To assess the accuracy of our approach, we applied the *MYD88* L265P ddPCR assay to cell-free DNA (cfDNA) reference specimens harboring the *MYD88* L265P mutation with a known AF. We used a series of commercially available reference samples containing several lymphoma-specific mutations including *MYD88* L265P with an AF of 1%, 0.5%, 0.1%, and WT (Seraseq® ctDNA Lymphoma Mix, Seracare). These samples were analyzed in triplicate and the median was determined. A PCC of at least 0.9 for the correlation of expected vs. measured AFs was considered acceptable for this performance parameter. **Supplementary Fig. S1E** shows a significant correlation between the median measured AF and the expected AF with a PCC of 0.99 ($p=0.002$).

Independent clinical validation of the assay and results of this validation approach

After assay development, the settings were locked and the *MYD88* L265P ddPCR assay was tested and validated in an independent clinical cohort of patients with contrast-enhancing brain lesions and verified histopathological diagnoses to evaluate its diagnostic performance parameters in both CSF and blood plasma samples. Samples were collected from patients participating in research studies conducted at the University Medical Center Freiburg, Germany (DRKS15307), and University Hospital of the Ludwig-Maximilians-University (LMU) Munich, Germany (Nr. 22-0008) that were approved by the ethics committees in accordance with the Declaration of Helsinki (see above). In total, we collected 77 CSF samples and 91 plasma samples from 87 CNSL patients and 41 Non-CNSL patients with a broad variety of malignant, inflammatory, and infectious brain diseases (**Supplementary Fig. S2A**). From 75 of these CSF/plasma samples and from 49 matched tumor specimens, the *MYD88* L265P mutation status was additionally assessed by a targeted-capture next-generation sequencing (NGS) technology that has been described in previous studies (CAPP-Seq, see below, **Supplementary Fig. S2A**)(1, 6, 7).

The 77 CSF specimens had a median volume of 1.9 mL (range: 0.2–9.9 mL), the 91 plasma samples a median volume of 8.5 mL (range: 2.7–11.8 mL, **Supplementary Fig. S2B**). They were collected from a total of 128 patients with contrast-enhancing brain lesions and verified histopathological diagnosis to evaluate its diagnostic sensitivity, specificity, positive predictive value (PPV), and negative predictive value (NPV) in both compartments (**Supplementary Fig. S2A, Supplementary Table 7**). Two-thirds of these patients (*n*=87, 68%) were diagnosed with either primary CNSL (PCNSL, 88%) or isolated secondary CNSL (iSCNSL, 12%) (defined as ‘CNSL

patients'), while 41 patients showed a broad variety of other brain diseases, including primary brain tumors (glioblastoma, lower-grade gliomas and others, 46%), brain metastases (25%), or inflammatory/infectious diseases such as multiple sclerosis (MS, 29%) (defined as 'Non-CNSL patients') (**Supplementary Fig. S2C, Supplementary Table 7**). In this setting, which is agnostic to the tumor mutation status ('tumor-agnostic approach'), we minimal-invasively detected *MYD88* L265P with a sensitivity of 67% from CSF and 37% from plasma samples of patients with confirmed CNSL (**Supplementary Fig. S3A**). In comparison, conventional CSF analyses including CP and FC identified CNSL infiltration with a sensitivity of 22% (n=46, **Supplementary Fig. S3B**). Importantly, the hotspot variant was never detected in Non-CNSL patients, revealing a specificity and PPV of 100% for both analytes (**Supplementary Fig. S3A**). The amount of ctDNA was 24-fold higher in *MYD88* L265P-positive CSF samples than in plasma, with a median CSF-ctDNA AF of 5.5% compared to 0.23% in blood plasma (p=0.0001, **Supplementary Fig. S3C**). In 49 CNSL cases, the *MYD88* L265P mutation was detected in a matched tumor specimen by targeted-capture NGS (**Supplementary Fig. S2A**). Limiting our analyses to CSF/plasma samples with corresponding tumors and known *MYD88* L265P positivity ('tumor-informed approach'), we observed a clinical sensitivity for our assay of 82% in CSF and 37% in plasma specimens (**Supplementary Fig. S3D**). Finally, we further assessed the reproducibility of our measurements and representation of allelic fractions across analytical platforms, comparing our results with those obtained from targeted-capture NGS of 75 CSF and plasma samples (**Supplementary Table 7**). We found a high concordance rate of 89.3% for the detection of *MYD88* L265P and a significant correlation of ctDNA concentrations between the technologies (p<0.0001,

r=0.99, **Supplementary Fig. S3E**). Collectively, these data demonstrate robust performance of the ddPCR technology in a representative clinical setting, revealing superior sensitivity for minimal-invasive *MYD88* L265P identification in the CSF compared to blood plasma, mainly due to markedly higher ctDNA concentrations in this compartment.

<u>Assay application in routine practice</u>

After assay development and independent validation, we implemented the approved *MYD88* L265P ddPCR technology in our clinical laboratory at the University Medical Center Freiburg, Germany, and prospectively analyzed CSF specimens obtained from hospitalized patients at 21 distinct centers between January 2022 and June 2024. Results of the analyses were reported directly to treating physicians, who made treatment decisions and conducted communication of findings with patients. A subset of patients (*n*=127), providing 143 CSF samples, participated in our observational study, approved by the ethics committee according to the Declaration of Helsinki, that facilitated the use of clinical, pathological, and radiological information, and enabled the assessment of clinical implications following CSF-ctDNA analyses (DRKS00034686, see above).

<u>Clinical data and analyses within the observational study</u>

For patients participating in the observational study, all relevant clinical information was available for this analysis for patients treated at the University Medical Center Freiburg. This included patient-specific, radiological, and pathological parameters. For

patients treated at external sites, we provided a questionnaire to the treating physician to obtain clinical information, outcome data, and the reason for the CSF submission to our laboratory to perform the analyses reported in this manuscript. In all patients except one, conventional CSF analyses including cytopathology (CP) and/or flow cytometry (FC) were performed as part of clinical routine to determine whether monoclonal lymphoma cells were present in the CSF compartment (**Supplementary Table 9**). A sample was considered positive when one of the two approaches or both showed infiltration of lymphoma cells. Magnetic resonance imaging (MRI) and/or computed tomography (CT) scans of the brain were performed as part of standard clinical care. Radiographic response of brain lesions was assessed according to the International PCNSL Collaborative Group (IPCG) criteria(8). Progression-free survival (PFS) was calculated from the lumbar puncture to lymphoma progression, relapse, or death from any cause. Overall survival (OS) was calculated from the lumbar puncture to death from any cause.

Statistical analyses and tests

To compare continuous variables, we used the non-parametric Mann Whitney U test. Linear relationships were determined using Pearson correlation (r). *P*-values <0.05 were considered as significant. Statistical tests were performed using GraphPad Prism (version 10.2.3).

Targeted next-generation sequencing

In addition to the ddPCR analyses, 75 CSF and plasma samples (**Supplementary Table 7**) as well as 49 CNSL tumor samples were simultaneously analyzed by targeted capture NGS using the Cancer Personalized Profiling by Deep Sequencing (CAPP-Seq) technology(1, 6, 9, 10). The NGS analyses have been performed as part of a previous publication and utilized for direct comparative evaluation of the presence of *MYD88* L265P between the ddPCR and the NGS platform(1). Specifically, to assess the reproducibility of our ddPCR measurements from patients of the independent validation cohort, we compared the presence of the *MYD88* L265P mutation and the allelic representation in all available 75 CSF and plasma samples between the two platforms (**Supplementary Fig. S3E**). Separately, we utilized the *MYD88* L265P genotyping information obtained by CAPP-Seq from CNSL tumor samples (*n*=49) to explore the ability of the ddPCR assay to detect the hotspot mutation in a 'tumor-informed' fashion, e.g. in patients with known *MYD88* L265P positivity (**Supplementary Fig. S3D**). Furthermore, this sensitive approach was also used to identify those *MYD88* L265P negative plasma samples used for the assessment of LOB (see above). All information on the CAPP-Seq approach and analysis of NGS data have been reported previously(1).

Supplementary Discussion

Due to the aggressiveness and localization of brain lymphomas, rapid diagnostic workup and treatment initiation are crucial to prevent neurological impairment and maintain favorable clinical outcomes(11). Yet, various factors in clinical reality, which can be either patient-specific, treatment-related, or associated with the disease, often impair a timely and accurate diagnosis of CNSL. In these situations, there is a critical

need for minimal-invasive approaches that utilize CSF biomarkers for the identification of CNSL. Current CNSL clinical guidelines such as the 2024 'EHA-ESMO Clinical Practice Guideline' consider the assessment of CSF biomarkers including *MYD88* L265P as a promising tool for CNSL diagnosis when biopsies are not possible(5). A plethora of research studies have explored protein and genetic markers for biopsy-free identification of CNSL from CSF and several laboratories have developed PCR-based assays for the detection of *MYD88* L265P from body fluids(1-3, 12-23). However, despite exhibiting some clinical utility in anecdotal cases and small CNSL case series(2, 23), these technologies have not been regularly and broadly applied in clinical routine yet and data demonstrating their value for clinical decision making and guiding treatment in large prospective patient cohorts are lacking. For example, Gupta et al. developed a rapid qPCR-based approach for the detection of mutations in *MYD88*, *TERT* promotor, *IDH1*, *IDH2*, *BRAF*, and *H3F3A* genes in CSF(2). While the detection of *MYD88* L265P influenced clinical decisions in 7 cases with brain lymphoma, the authors noted that 'larger studies are needed to determine whether their findings in a limited number of patients are broadly applicable'(2).

We here underwent an extensive regulatory development and validation process that led to the implementation of a *MYD88* L265P ddPCR assay in our clinical laboratory environment for minimal-invasive detection of CNSL from CSF. This procedure was optimized for high specificity to minimize false-positive results and misclassification. In an extensive validation procedure before clinical implementation, we did not find *MYD88* L265P in the CSF of patients with contrast-enhancing CNS lesions and verified Non-CNSL diagnosis, translating to a specificity and PPV of 100%. However, *MYD88* L265P mutations have been associated with other brain diseases and

conditions, which necessitates careful interpretation in the specific clinical context. For example, *MYD88* L265P can be detected in most patients with Bing-Neel syndrome (BNS), a rare disease manifestation of indolent Waldenstrom macroglobulinemia in the CNS(24). Indeed, BNS cannot be distinguished from aggressive CNSL by the detection of *MYD88* L265P from CSF alone and correlations with radiological and clinical parameters need to be considered. Therapeutic strategies for BNS are not standardized and heterogeneous; however, treatment of patients with symptomatic BNS are often adapted from those used for aggressive PCNSL, including high-dose methotrexate and cytarabine(25). Separately, one previous publication has described the detection of *MYD88* L265P in two patients with suspected MS and anti-GFAP astrocytopathy, where the conditions were diagnosed by CSF analyses without definitive exclusion of CNSL through stereotactic biopsies(26). In our study and in work from other groups that systematically investigated *MYD88* L265P in CSF, the hotspot mutation was never observed in patients with confirmed inflammatory or autoimmune brain diseases, rendering misdiagnosis a highly unlikely scenario(2, 27). Finally, *MYD88* L265P mutations can be found in the blood of individuals with clonal hematopoiesis of indeterminate potential (CHIP) and IgM-MGUS(28, 29). However, the clinical scenarios investigated in our study differ significantly from these conditions, as patients with CHIP and IgM-MGUS are largely asymptomatic. Furthermore, and most importantly, our clinical laboratory analyzes CSF in the routine setting and not blood, making a misclassification involving these conditions without the presence of brain lymphoma extremely unlikely.

We applied our LDT to 205 CSF samples over a period of 2.5 years in clinical practice and assessed its implications in an observational study. Results were communicated

to treating physicians within 5 days (including weekends) and directly guided or helped guiding clinical management in 60% of CSF-ctDNA positive cases. Neurosurgical interventions were obviated or CNSL diagnosis was accelerated in 19 patients and a treatment-guiding effect was reported for 25 patients who received CNS-directed agents based on or supported by minimal-invasive CSF-ctDNA detection. Importantly, all but one patient objectively responded to CNSL-specific therapies, illustrating the clinical benefit of our liquid biopsy approach for a significant subgroup of patients whose final diagnosis otherwise might have remained unconfirmed or who would have faced high-risk surgical interventions to obtain a diagnosis. Thus, compared to previous publications in the field, the key advancement of our study is the prospective demonstration that minimal-invasive identification of CNSL can effectively guide clinical decisions and treatment, ultimately benefiting patients in routine practice and within a large patient cohort. Yet, in contrast to other studies, the real-world patient cohort we assessed in our study was rather heterogeneous, including patients with lymphoma entities such as Burkitt lymphoma or mantle cell lymphoma, in which *MYD88* L265P is not expected. Furthermore, some patients with B-cell lymphomas who were asymptomatic for CNS involvement showed a positive CSF-ctDNA result in our observational study (**Supplementary Table S9**). In these cases, the optimal treatment is still unclear and warrants further investigation in prospective clinical trials.

Of note, while positive CSF-ctDNA results can directly guide treatment or surgical strategies due to their high PPV, assessing the clinical impact of CSF-ctDNA negative results represents a considerable challenge, because a negative *MYD88* L265P status does not exclude CNSL or help defining another diagnosis. Thus, knowing that CSF samples are negative for *MYD88* L265P might help narrow down differential

diagnoses in certain scenarios, but it typically has no direct influence on the clinical management of patients with unknown CNS lesions.

Another notable finding of our study was that the exposure to corticosteroids prior to lumbar punctures did not seem to have any impact on CNSL detection rates or the representation of *MYD88* L265P allelic fractions. Corticosteroids are often used to manage neurological symptoms in CNSL patients but cause severe diagnostic delays and other challenges associated with steroid-induced transient lymphoma regression(30, 31). Our results indicate that CSF-ctDNA can be robustly detected irrespective of corticosteroid premedication, encouraging the use of minimal-invasive liquid biopsy technologies even in situations with radiological tumor reduction following steroid therapy. Yet, the duration of corticosteroid treatment and dosage were highly heterogeneous in our patient cohort, introducing some uncertainties around interpretability of the results and requiring further investigation of the corticosteroid effect for CSF-ctDNA detection in a prospective and standardized fashion.

Another limitation of our technology can be attributed to the fact that approximately 30% of CNSL patients do not harbor the hotspot *MYD88* L265P mutation, inevitably reducing applicability and sensitivity/NPV of our tumor-agnostic approach(32). Alternative strategies have revealed higher sensitivities for CNSL identification, including assays that combine genetic and protein biomarkers or next-generation sequencing-based technologies. However, these methods require further regulatory standardization and have yet to prove their utility in clinical practice and large patient cohorts(1, 3, 12, 23).

Supplementary Figures

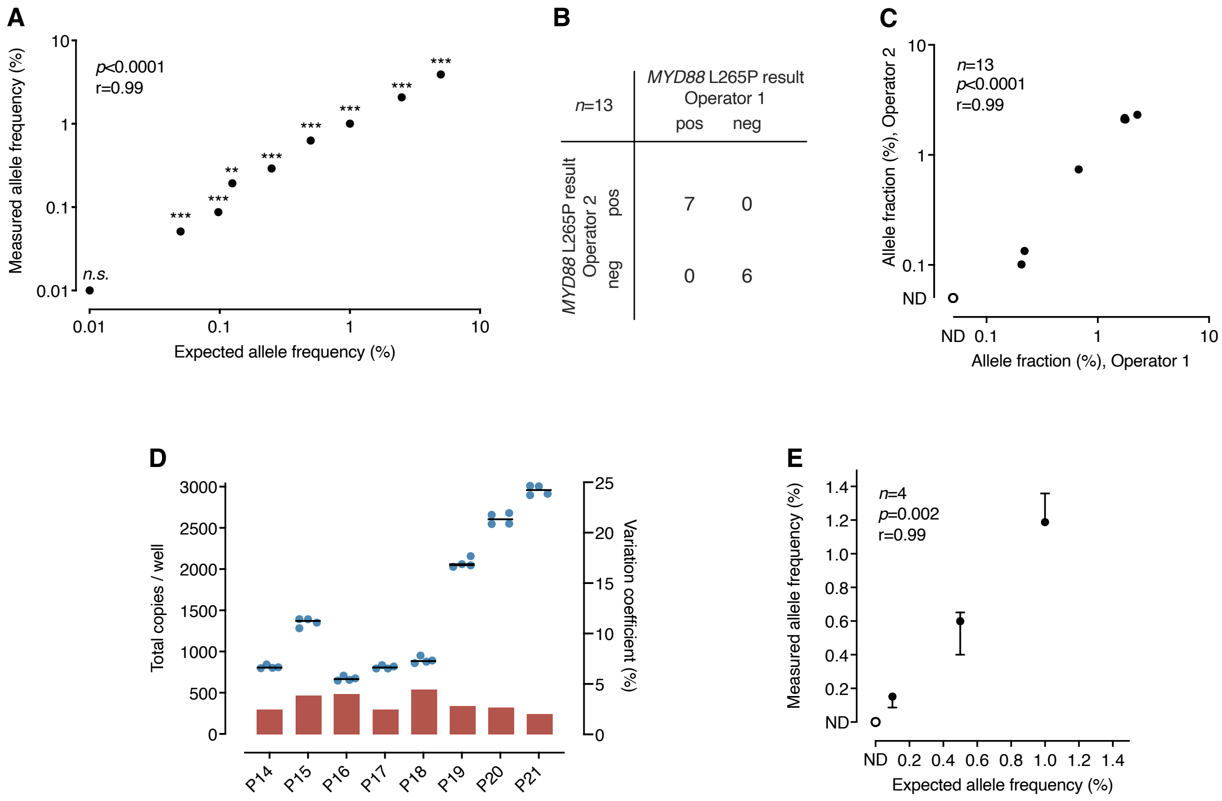

Supplementary Fig. S1. Performance parameters of the development workflow for the *MYD88* L265P ddPCR assay. (A) Assessing the limit of detection (LOD) by serial dilutions of recombinant mutant DNA fragments spiked into human genomic DNA with concentrations of 1:20 (5%), 1:40 (2.5%), 1:100 (1%), 1:200 (0.5%), 1:400 (0.25%), 1:800 (0.125%), 1:1,000 (0.1%), 1:2,000 (0.05%), and 1:10,000 (0.01%). Shown is the correlation of the expected allele frequency on the x-axis and the measured allele frequency as the average of 4 measurements on the y-axis. Asterisks on top of the black dots indicate whether the signal of the respective dilution was significantly above the background. **(B,C)** Assessment of inter-assay reliability. **(B)** Contingency table displaying the results of *MYD88* L265P testing by two independent operators in 13 plasma samples from CNSL patients with known *MYD88* L265P status and **(C)** correlation of ctDNA allele frequencies between measurements performed by

operator 1 (x-axis) and operator 2 (y-axis). **(D)** Assessment of intra-assay reliability by comparing the number of DNA copies detected across single wells within the *MYD88* L265P assay. Four technical replicates of eight plasma samples from CNSL patients were investigated, calculating the sum of mutant and wildtype copies per well (blue dots, left y-axis, black line: median). Variation coefficients (in %) of these replicates for each of the eight cases are depicted on the right y-axis. **(E)** Assessment of accuracy. The ddPCR assay was applied to cell-free DNA reference specimens harboring known concentrations of the *MYD88* L265P mutation. Commercially available cfDNA reference samples containing the *MYD88* L265P mutation with allele frequencies of 1%, 0.5%, 0.1%, and 0% (wildtype) were analyzed. Shown here is the correlation of expected allele frequencies on the x-axis and measured allele frequencies on the y-axis. Black dots represent the median and black lines the range of of triplicate measurement for each dilution. *n.s.*, not significant; pos, positive; neg, negative; ND, not detected.

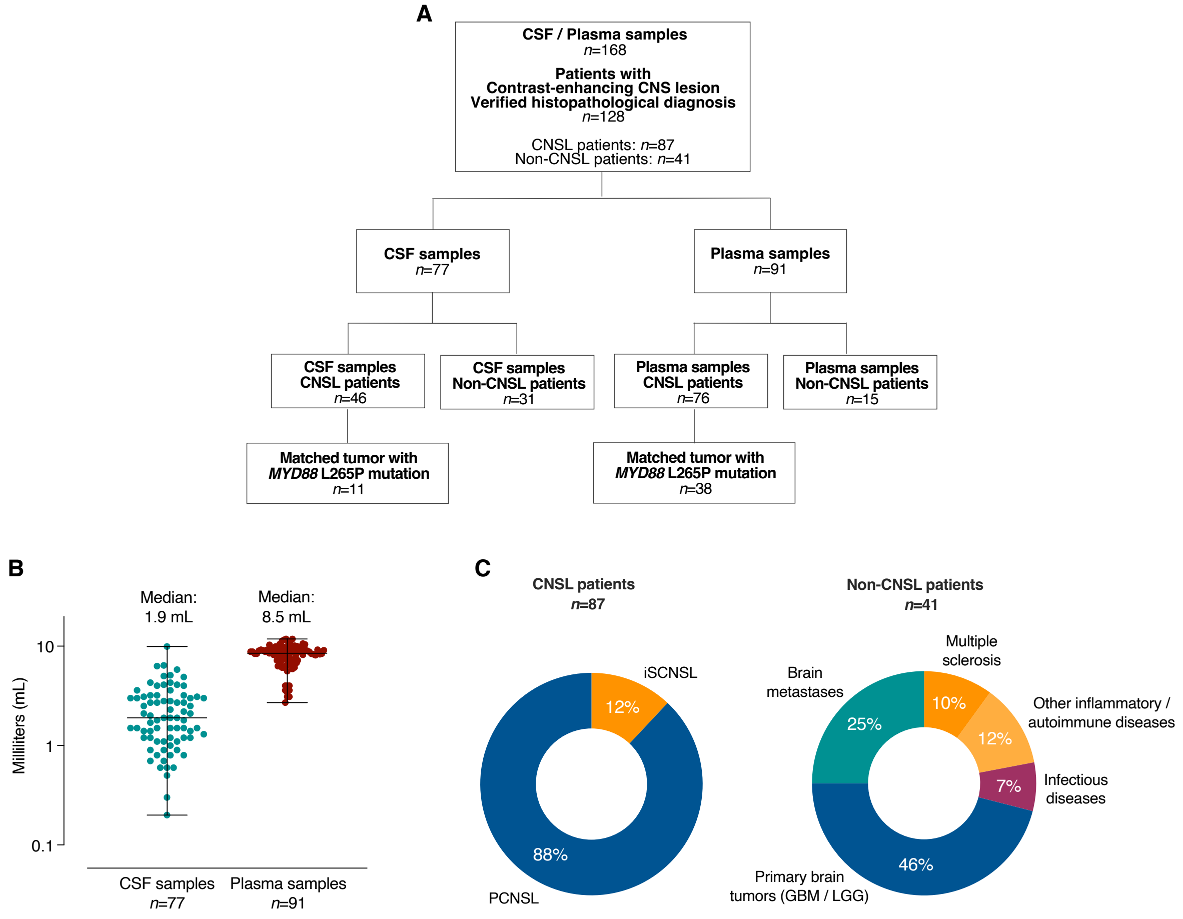

Supplementary Fig. S2. Independent validation of the *MYD88* L265P ddPCR assay. (A) Overview and consort diagram showing patients and specimens available from the independent validation cohort. **(B)** Scatter plots showing the CSF (left) and plasma sample volumes (right) available for *MYD88* L265P testing in the independent validation cohort. Black lines indicate the median and range of sample volumes. **(C)** Pie charts depicting the distribution of brain diseases in the independent validation cohort. mL, milliliters; CSF, cerebrospinal fluid; CNS, central nervous system; CNSL, CNS lymphoma; PCNSL, primary CNSL; iSCNSL, isolated secondary CNSL; GBM, glioblastoma; LLG, lower grade gliomas.

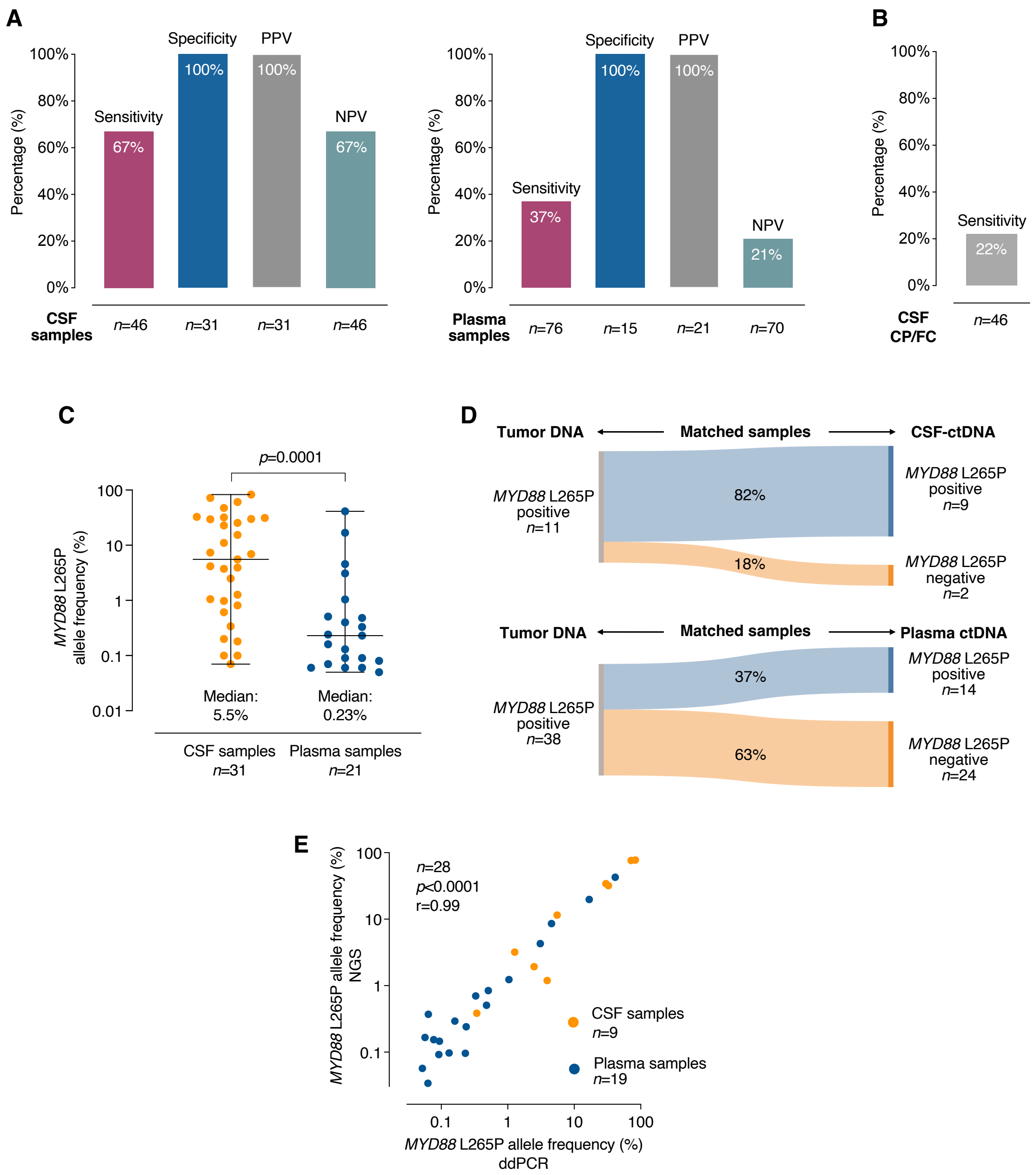

Supplementary Fig. S3. ddPCR assay performance in the independent validation cohort. (A) Performance parameters of the *MYD88* L265P assay applied to CSF (left) and plasma samples (right) from patients of the independent validation cohort in a tumor-agnostic setting. **(B)** Sensitivity of CNSL detection by conventional CSF cytology and flow cytometry analyses in the independent validation cohort, depicted as a bar graph. **(C)** Concentrations of ctDNA (allele frequencies, in percent) measured

by *MYD88* L265P analysis from CSF (left) and plasma (right). Black lines represent the median and range of allele frequencies. **(D)** Sankey plots showing the *MYD88* L265P positivity rate in CSF (top) and plasma (bottom) from CNSL patients with known *MYD88* L265P-positive tumors ('tumor-informed approach'). **(E)** Comparison of *MYD88* L265P allele frequencies measured either by the ddPCR assay (x-axis) or the targeted NGS platform (y-axis) from CSF (orange dots) or plasma (blue dots). CSF, cerebrospinal fluid; CP, cytopathology; FC, flow cytometry; NGS, next-generation sequencing; ddPCR, digital droplet PCR; PPV, positive predictive value; NPV, negative predictive value.

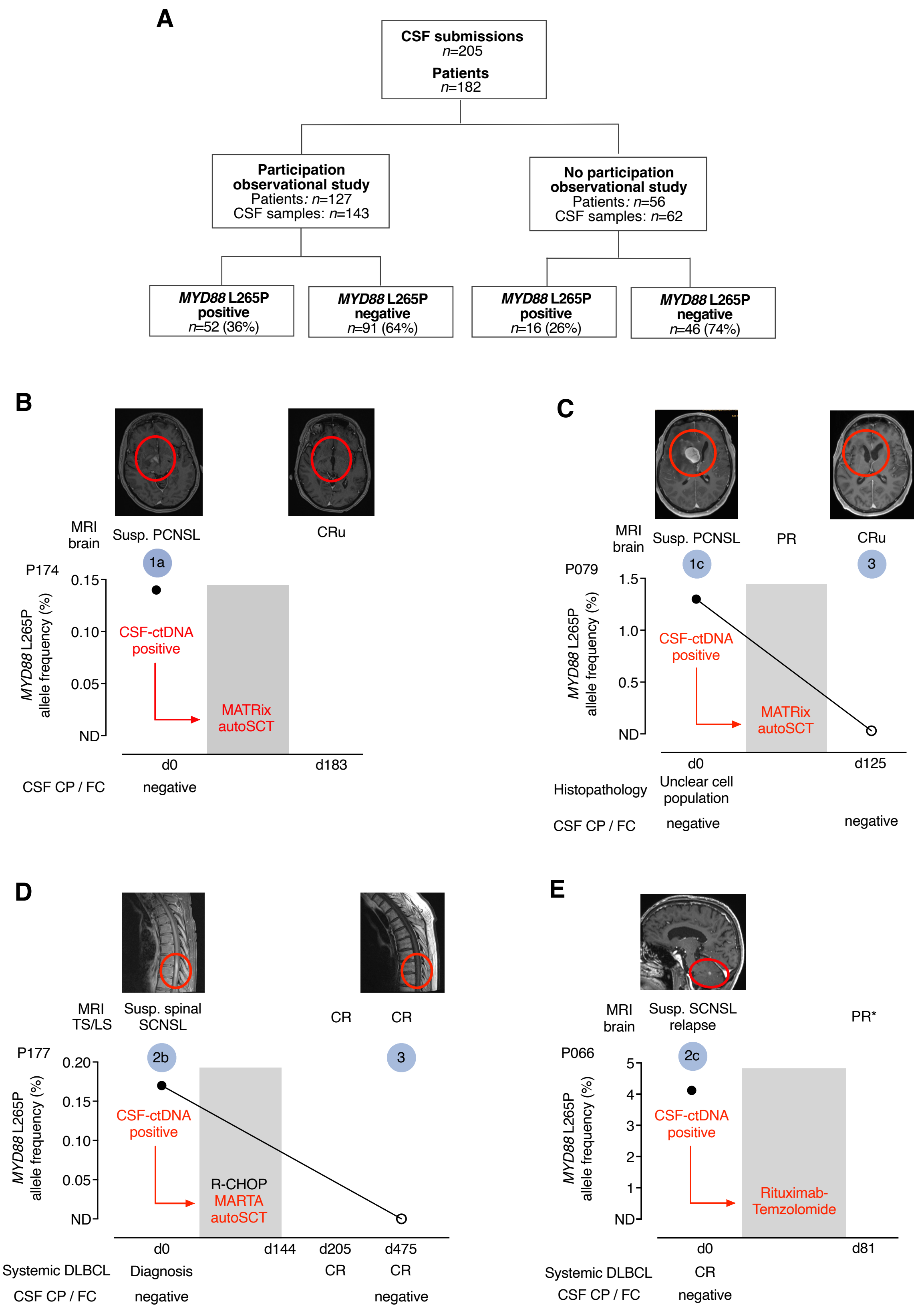
A
CSF submissions
n=205
Patients
n=182
Participation observational study
Patients: n=127
CSF samples: n=143
No participation observational study
Patients: n=56
CSF samples: n=62
MYD88 L265P positive
n=52 (36%)
MYD88 L265P negative
n=91 (64%)
MYD88 L265P positive
n=16 (26%)
MYD88 L265P negative
n=46 (74%)
B
MRI brain
Susp. PCNSL
CRu
P174
1a
MYD88 L265P allele frequency (%)
0.15
0.10
0.05
ND
CSF-ctDNA positive
MATRix autoSCT
d0
d183
CSF CP / FC
negative
C
MRI brain
Susp. PCNSL
PR
CRu
P079
1c
3
MYD88 L265P allele frequency (%)
1.5
1.0
0.5
ND
CSF-ctDNA positive
MATRix autoSCT
d0
d125
Histopathology
Unclear cell population
CSF CP / FC
negative
negative
D
MRI TS/LS
Susp. spinal SCNSL
CR
CR
P177
2b
3
MYD88 L265P allele frequency (%)
0.20
0.15
0.10
0.05
ND
CSF-ctDNA positive
R-CHOP
MARTA
autoSCT
d0
d144
d205
d475
Systemic DLBCL
Diagnosis
CR
CR
CSF CP / FC
negative
negative
E
MRI brain
Susp. SCNSL relapse
PR*
P066
2c
MYD88 L265P allele frequency (%)
5
4
3
2
1
ND
CSF-ctDNA positive
Rituximab-Temzolomide
d0
d81
Systemic DLBCL
CR
CSF CP / FC
negative

Supplementary Fig. S4. Clinical implementation of the *MYD88* L265P ddPCR assay and its implications for individual patient management. (A) Overview and consort diagram showing the submitted CSF samples for routine *MYD88* L265P analysis and patient participation in the observational study. **(B)** Timeline depicting the course of a 67-year-old female patient who was diagnosed with a contrast-enhancing lesion in the right basal ganglia by MRI. Stereotactic biopsy was considered high-risk due to the localization and risk of bleeding. CSF was submitted to our laboratory for *MYD88* L265P testing and we reported a positive result back (0.14% allele frequency) with a turnaround time of 5 days. The patient received 3 cycles of high-dose methotrexate-based induction chemotherapies (MATRix) followed by high-dose chemotherapy and autologous stem cell transplantation solely based on the CSF-ctDNA results. After completion of treatment, MRI revealed an unconfirmed complete remission. **(C)** Shown is a timeline of a 55-year-old male patient who was admitted to the hospital with headaches and hemiparesis of the left arm. MRI revealed a contrast-enhancing lesion in the right rostral striatum/frontal lobe, suspicious for CNSL. A stereotactic biopsy was performed and the tumor assessed by histopathology. The final diagnosis remained unconfirmed with evidence of unclear desmoplastic tissue fragments. CSF from this patient was submitted for ctDNA analysis and the *MYD88* L265P mutation was identified with an allele frequency of 1.3%, while conventional CSF analyses were negative for monoclonal lymphoma cells. We reported the result back to the treating physician within 1 day. Based on the CSF-ctDNA result, patient received 2 cycles of MATRix followed by high-dose chemotherapy and autologous stem cell transplantation. End-of-treatment MRI showed an unconfirmed complete response according to the IPCG criteria and *MYD88* L265P was undetectable in CSF

after completion of therapy. **(D)** In a 75-year-old male patient, DLBCL was diagnosed after resection of an abdominal lymph node. Due to incontinence, an MRI of the thoracic (shown in the figure panel) and lumbar spine was performed, revealing two contrast-enhancing lesions, suspected for synchronous secondary CNS involvement of DLBCL. A lumbar puncture was performed and CSF submitted for ctDNA analysis as well as CP/FC analyses. While CP/FC showed no definitive result, *MYD88* L265P was identified with an allele frequency of 0.17%, supporting the diagnosis of secondary CNSL. The patient received R-CHOP immunochemotherapy and the CNS-directed MARTA protocol followed by high-dose chemotherapy and autologous stem cell transplantation. While the neurological symptoms remained after treatment, radiological response assessment revealed complete remission for both the systemic DLBCL and SCNSL, supported by undetectable CSF-ctDNA more than one year after stem cell transplantation. **(E)** Timeline depicting the course of an 80-year-old patient with a history of SCNSL that was in complete remission after methotrexate-based immunochemotherapy and autologous stem cell transplantation. The patient presented with disorientation two years after transplant; an MRI showed a cerebellar contrast-enhancing lesion suspected for metachronous SCNSL relapse. Due to frailty, a neurosurgical intervention was not performed. CSF-ctDNA analysis showed a *MYD88* L265P mutation with an allele frequency of 4.12%, reported to the treating physician with a turnaround time of one day. A palliative CNS-directed therapy was initiated after exclusion of systemic DLBCL relapse with rituximab and temozolomide. Patient's condition improved rapidly after initiation of therapy and a follow-up MRI showed a partial response 81 days after start of treatment. Asterisk: no image available from radiological follow-up assessment. CSF, cerebrospinal fluid; MRI,

magnetic resonance imaging; PCNSL, primary central nervous system lymphoma; SCNSL, secondary CNSL; Susp., suspected; CP, cytopathology; FC, flow cytometry; d, day; CR, complete response; CRu, unconfirmed complete response; ND, not detected; PR, partial response; ctDNA, circulating tumor DNA; DLBCL, diffuse large B-cell lymphoma; LS, lumbosacral; autoSCT, autologous stem cell transplantation; MATRix, high-dose methotrexate, high-dose cytarabine, thiotepa, rituximab; R-CHOP, rituximab, cyclophosphamide, doxorubicin, vincristine, prednisolone; MARTA, high-dose methotrexate, high-dose cytarabine, rituximab.

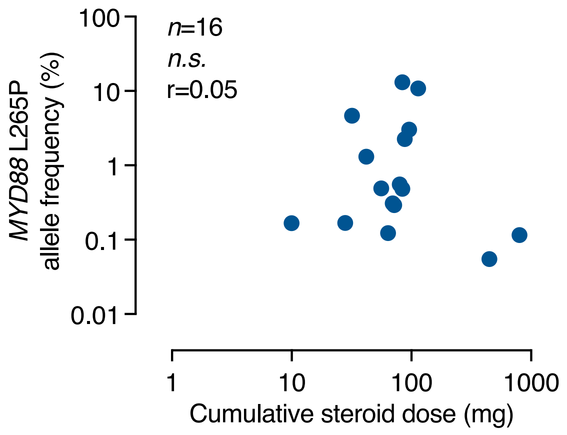

Supplementary Fig. S5. Association of the cumulative steroid dose and *MYD88* L265P AFs. Scatter plot comparing the cumulative corticosteroid dose on the x-axis with *MYD88* L265P allele frequencies in CSF-ctDNA positive cases (y-axis). mg, milligram.

References

1. Mutter JA, Alig SK, Esfahani MS, Lauer EM, Mitschke J, Kurtz DM, et al. Circulating Tumor DNA Profiling for Detection, Risk Stratification, and Classification of Brain Lymphomas. J Clin Oncol. 2023;41(9):1684-94.

2. Gupta M, Bradley J, Massaad E, Burns E, Georgantas NZ, Maron G, et al. Rapid Tumor DNA Analysis of Cerebrospinal Fluid Accelerates Treatment of Central Nervous System Lymphoma. Blood. 2024.

3. Bravetti C, Degaud M, Armand M, Sourdeau E, Mokhtari K, Maloum K, et al. Combining MYD88 L265P mutation detection and clonality determination on CSF cellular and cell-free DNA improves diagnosis of primary CNS lymphoma. Br J Haematol. 2023;201(6):1088-96.

4. Armbruster DA, Pry T. Limit of blank, limit of detection and limit of quantitation. Clin Biochem Rev. 2008;29 Suppl 1(Suppl 1):S49-52.

5. Ferreri AJM, Illerhaus G, Doorduijn JK, Auer DP, Bromberg JEC, Calimeri T, et al. Primary central nervous system lymphomas: EHA-ESMO Clinical Practice Guideline for diagnosis, treatment and follow-up. Hemasphere. 2024;8(6):e89.

6. Scherer F, Kurtz DM, Newman AM, Stehr H, Craig AF, Esfahani MS, et al. Distinct biological subtypes and patterns of genome evolution in lymphoma revealed by circulating tumor DNA. Sci Transl Med. 2016;8(364):364ra155.

7. Kurtz DM, Scherer F, Jin MC, Soo J, Craig AFM, Esfahani MS, et al. Circulating Tumor DNA Measurements As Early Outcome Predictors in Diffuse Large B-Cell Lymphoma. J Clin Oncol. 2018;36(28):2845-53.

8. Abrey LE, Batchelor TT, Ferreri AJ, Gospodarowicz M, Pulczynski EJ, Zucca E, et al. Report of an international workshop to standardize baseline evaluation and response criteria for primary CNS lymphoma. J Clin Oncol. 2005;23(22):5034-43.

9. Newman AM, Lovejoy AF, Klass DM, Kurtz DM, Chabon JJ, Scherer F, et al. Integrated digital error suppression for improved detection of circulating tumor DNA. Nat Biotechnol. 2016;34(5):547-55.

10. Newman AM, Bratman SV, To J, Wynne JF, Eclov NC, Modlin LA, et al. An ultrasensitive method for quantitating circulating tumor DNA with broad patient coverage. Nat Med. 2014;20(5):548-54.

11. Cerqua R, Balestrini S, Perozzi C, Cameriere V, Renzi S, Lagalla G, et al. Diagnostic delay and prognosis in primary central nervous system lymphoma compared with glioblastoma multiforme. Neurol Sci. 2016;37(1):23-9.

12. Ferreri AJM, Calimeri T, Lopedote P, Francaviglia I, Daverio R, Iacona C, et al. MYD88 L265P mutation and interleukin-10 detection in cerebrospinal fluid are highly specific discriminating markers in patients with primary central nervous system lymphoma: results from a prospective study. Br J Haematol. 2021;193(3):497-505.

13. Schroers R, Baraniskin A, Heute C, Vorgerd M, Brunn A, Kuhnhenn J, et al. Diagnosis of leptomeningeal disease in diffuse large B-cell lymphomas of the central nervous system by flow cytometry and cytopathology. Eur J Haematol. 2010;85(6):520-8.

14. Baraniskin A, Schroers R. Liquid Biopsy and Other Non-Invasive Diagnostic Measures in PCNSL. Cancers (Basel). 2021;13(11).

15. Rimelen V, Ahle G, Pencreach E, Zinniger N, Debliquis A, Zalmaï L, et al. Tumor cell-free DNA detection in CSF for primary CNS lymphoma diagnosis. Acta Neuropathol Commun. 2019;7(1):43.

16. Olszewski AJ, Chorzalska AD, Petersen M, Ollila TA, Zayac A, Kurt H, et al. Detection of clonotypic DNA in the cerebrospinal fluid as a marker of central nervous system invasion in lymphoma. Blood Adv. 2021;5(24):5525-35.

17. Sasayama T, Nakamizo S, Nishihara M, Kawamura A, Tanaka H, Mizukawa K, et al. Cerebrospinal fluid interleukin-10 is a potentially useful biomarker in immunocompetent primary central nervous system lymphoma (PCNSL). Neuro Oncol. 2012;14(3):368-80.

18. Rubenstein JL, Wong VS, Kadoch C, Gao HX, Barajas R, Chen L, et al. CXCL13 plus interleukin 10 is highly specific for the diagnosis of CNS lymphoma. Blood. 2013;121(23):4740-8.

19. Hiemcke-Jiwa LS, Leguit RJ, Snijders TJ, Bromberg JEC, Nierkens S, Jiwa NM, et al. MYD88 p.(L265P) detection on cell-free DNA in liquid biopsies of patients with primary central nervous system lymphoma. Br J Haematol. 2019;185(5):974-7.

20. Hiemcke-Jiwa LS, Minnema MC, Radersma-van Loon JH, Jiwa NM, de Boer M, Leguit RJ, et al. The use of droplet digital PCR in liquid biopsies: A highly sensitive technique for MYD88 p.(L265P) detection in cerebrospinal fluid. Hematol Oncol. 2018;36(2):429-35.

21. Watanabe J, Natsumeda M, Kanemaru Y, Okada M, Oishi M, Kakita A, et al. Comparison of circulating tumor DNA between body fluids in patients with primary central nervous system lymphoma. Leuk Lymphoma. 2019;60(14):3587-9.

22. Yamagishi Y, Sasaki N, Nakano Y, Matushita Y, Omura T, Shimizu S, et al. Liquid biopsy of cerebrospinal fluid for MYD88 L265P mutation is useful for diagnosis of central nervous system lymphoma. Cancer Sci. 2021;112(11):4702-10.

23. Calimeri T, Anzalone N, Cangi MG, Fiore P, Gagliardi F, Miserocchi E, et al. Molecular diagnosis of primary CNS lymphoma in 2024 using MYD88. Lancet Haematol. 2024;11(7):e540-e9.

24. Treon SP, Xu L, Yang G, Zhou Y, Liu X, Cao Y, et al. MYD88 L265P somatic mutation in Waldenström's macroglobulinemia. N Engl J Med. 2012;367(9):826-33.

25. Minnema MC, Kimby E, D'Sa S, Fornecker LM, Poulain S, Snijders TJ, et al. Guideline for the diagnosis, treatment and response criteria for Bing-Neel syndrome. Haematologica. 2017;102(1):43-51.

26. Oetjen LK, Bhattacharyya S, Galetta K. MYD88 L265P mutation in neurologic autoimmunity without evidence of malignancy. Mult Scler Relat Disord. 2023;77:104868.

27. Gupta M, Burns E, Georgantas NZ, Thierauf J, Nayyar N, Gordon A, et al. A Rapid Genotyping Panel for Detection of Primary Central Nervous System Lymphoma. Blood. 2021.

28. Niroula A, Sekar A, Murakami MA, Trinder M, Agrawal M, Wong WJ, et al. Distinction of lymphoid and myeloid clonal hematopoiesis. Nat Med. 2021;27(11):1921-7.

29. Varettoni M, Arcaini L, Zibellini S, Boveri E, Rattotti S, Riboni R, et al. Prevalence and clinical significance of the MYD88 (L265P) somatic mutation in Waldenstrom's macroglobulinemia and related lymphoid neoplasms. Blood. 2013;121(13):2522-8.

30. Deckert M, Brunn A, Montesinos-Rongen M, Terreni MR, Ponzoni M. Primary lymphoma of the central nervous system--a diagnostic challenge. Hematol Oncol. 2014;32(2):57-67.

31. Bromberg JE, Siemers MD, Taphoorn MJ. Is a "vanishing tumor" always a lymphoma? Neurology. 2002;59(5):762-4.

32. Hernández-Verdin I, Kirasic E, Wienand K, Mokhtari K, Eimer S, Loiseau H, et al. Molecular and clinical diversity in primary central nervous system lymphoma. Ann Oncol. 2022.
